# Phenotypic spectrum and mechanism analysis of Schaff Yang syndrome: A case report on new mutation of MAGEL2 gene

**DOI:** 10.1097/MD.0000000000026309

**Published:** 2021-06-18

**Authors:** Yanjie Duan, Lu Liu, Xiujuan Zhang, Xiuyun Jiang, Jin Xu, Qingbo Guan

**Affiliations:** a Department of Endocrinology, Shandong Provincial Hospital affiliated to Shandong First Medical University; b Department of Endocrinology, Shandong Provincial Key Laboratory of Endocrinology and Lipid Metabolism; c Department of Endocrinology, Shandong Provincial Hospital Affiliated to Shandong First Medical University, Jinan, Shandong, China.

**Keywords:** case report, melanoma antigen L2, phenotype, Prader-Willi syndrome, Schaaf-Yang syndrome

## Abstract

**Rationale:**

The Schaaf-Yang syndrome (SYS) is an autosomal dominant multi-system genetic disease caused by melanoma antigen L2 (MAGEL2) gene mutations imprinted by mothers and expressed by fathers on the 15q11–15q13 chromosomes in the critical region of Prader-Willi. MAGEL2 is a single exon gene and one of the protein-coding genes of the Prader-Willi domain. MAGEL2 is a matrilineal imprinted gene (i.e., the maternal chromosome is methylated). It is only expressed by unmethylated paternal alleles, and the individual is affected only when the variation occurs on the paternal allele.

**Patient concerns:**

We reported a patient with MAGEL2 gene new site mutation who had mild intellectual disability, social fear, small hands and feet, obesity issues, dyskinesia, growth retardation, language lag and sexual development disorder.

**Diagnosis:**

Whole-exome sequencing showed a heterozygous variation in the MAGEL2 gene, NM_019066.4:c.1687C > T (p.Q563X) and diagnosed as Schaaf-Yang syndrome.

**Interventions:**

Patient was advised to reduce weight, control blood lipids, blood glucose through appropriate strengthening of exercise and diet control in the future. At the same time, the family members were advised to provide mental training to the patient to strengthen the contact and communication with the outside world and improve the autistic symptoms. Because of the patient's bilateral cryptorchidism, it is recommended that the patient should be treated with bilateral cryptorchidism reduction fixation.

**Outcomes:**

After a follow-up of the patient for 2 months, the patient is still walking unsteadily and requires an auxiliary reference material to walk normally. There is no significant change in height compared to before, and the weight has dropped by about 2 kg in the past 2 months. The symptoms of autism have improved slightly. The patient is willing to communicate with outsiders; his intelligence has not improved significantly, and his academic performance in school is still at the middle and lower levels.

**Lessons:**

The pathogenesis of SYS is complex, involving multiple pathways such as Leptin-POMC, MAGEL2-USP7-TRIM27 complex and oxytocin. Our study has also found that certain fatal phenotypes such as respiratory distress have a high incidence at individual sites, and early detection and timely intervention may prolong the life span of patients. Therefore, for patients in whom SYS is highly suspected, gene detection should be carried out as soon as possible.

## 1 Introduction

Schaaf-Yang syndrome (SYS) (OMIM 615547) is an autosomal dominant multi-system genetic disease caused by Melanoma antigen L2 (MAGEL2) gene mutations imprinted by mothers and expressed by fathers on 15q11–15q13 chromosomes in the critical region of Prader-Willi. MAGEL2 is a single exon gene and one of the protein-coding genes of the Prader-Willi domain. This gene is a member of the MAGE family of ubiquitin ligase regulators, which encodes a protein that is important for protein transport in the body.^[1,2]^ Given that MAGEL2 is a matrilineal imprinted gene (i.e., the maternal chromosome is methylated), it is only expressed by unmethylated paternal alleles, and the individual is affected only when the variation occurs on the paternal allele. Based on our research, we found that the characteristic phenotype of SYS is developmental delay and intellectual disability, hypotonia and abnormal behavior. The other features include contractures, feeding difficulties, autism spectrum disorder, and dysmorphic facial features. Interestingly, 4 SYS patients with truncated mutations in paternal copies of MAGEL2, who were reported for the first time in 2013, were initially considered to be patients of the “Prader-Willi syndrome (PWS)”. In addition to the location of the MAGEL2 gene in the Prader-Willi domain, the phenotypic overlap of SYS and PWS is also an important factor. This paper discusses the phenotype, gene locus and some mechanisms of MAGEL2 in a patient with an SYS gene mutation that has not been reported before.

## 2 Case presentation

The patient, a teenage male, 16 years old, mainly having a short penis for (16 years), was suffering from progressive weight gain (for 9 years), unstable walking (for more than 1 month). The mother did not take drugs during her pregnancy, and there was no history of exposure to any poisonous and radioactive substances. He is an off-spring of non-consanguineous parents, born full-term normal delivery, with cephalic presentation, no history of hypoxia, birth weight 3.3 kg, no complications such as obvious hypotonia and feeding difficulties after birth (The birth length is not known). The child was breastfed till he turned 2 years old, and while he started walking at 2 years old, he commenced talking later than his peers. The child also showed slow reaction since childhood, poor academic performance, mild intellectual disability and social fear. Movement disorder was noted in July 2020, needing a visual reference when he walks, otherwise he walked unsteadily. The patient was 155 cm tall and weighed 105 kg, and his BMI was, therefore, 43.70 kg/m^2^. According to the percentile curve of height and weight of boys aged 2 to 18 years old obtained from the physical development survey of 9 provinces and cities in China in 2005, the patient's height is less than 3% of the same age and sex in the same region, and his weight is more than 97%. Further, he had thick and black hair with a thick posterior hairline to the neck, thick eyebrows, wide eye-distance, collapsed nose, some beard scattered around the mouth, no Adam's apple, changed voice, armpit hair, and smaller hands and feet when compared to his peers. There was no breast development on either side. He had a thick vulva and pubic hair and pigmentation in the penis and scrotum, but there had been no obvious enlargement of his penis since he turned 9 years old, no morning erection and no spermatorrhea. The penis was found to be about 4 cm long in the non-erected state, and the left testicle's volume was found to be about 1 mL. The right testis was not palpable.

Based on the patient's medical history, imaging examination and molecular genetic testing results, the diagnosis was SYS. Patient was advised to reduce weight, control blood lipids, blood glucose through appropriate strengthening of exercise and diet control in the future. At the same time, the family members were advised to provide mental training to the patients to strengthen their contact and communication with the outside world and improve the autistic symptoms of the patient. Because of the patient's bilateral cryptorchidism, it is recommended that the patient should be treated with bilateral cryptorchidism reduction fixation in the department of urology. After a follow-up of the patient for 2 months, the patient is still walking unsteadily and requires an auxiliary reference material to walk normally. There is no significant change in height compared to before, and the weight has dropped by about 2 kg in the past 2 months. The rest of the physical examination did not show significant changes. The symptoms of autism have improved slightly. The patient is willing to communicate with outsiders, his intelligence has not improved significantly, and his academic performance in school is still at the middle and lower levels.

## 3 Genetic analysis – methods

After obtaining the informed consent from the patient's parents, genomic DNA was extracted from peripheral blood samples of the patient, and whole-exome sequencing was performed. After the genomic DNA extracted from the samples was segmented, ligated, amplified and purified, the DNA library was prepared by hybridisation capture, and then the exon region and flanking intron region (20 bp) of 20,099 genes in the human exon group were detected using the high-throughput sequencing platform. The sequencing data were compared with the human genome hg19 (GRCh37) reference sequence, and the coverage and sequencing quality of the target region were evaluated. The pathogenicity of the variation was assessed according to the guidelines of the American College of Medical Genetics and Genomics (ACMG) published in 2015. This test only reports those variants that are related to the patient's phenotype or test results and are classified as pathogenic, probable or clinically ambiguous. When the pathogenic or possible pathogenic variation that is detected is in the autosomal recessive gene, the laboratory ensures that the coverage of the gene coding sequence reaches 100% by NGS and/or Sanger sequencing. The clinical investigations and genetic analyses were approved by the Medical Ethical Committee of Shandong Provincial Hospital Affiliated to Shandong First Medical University.

## 4 Result of the genetic analysis

A heterozygous variation, NM_019066.4:c.1687C > T (p.Q563X), was found in the patient's MAGEL2 gene, which has not been reported in the existing literature and the following gene database BIOGPS, GeneCards, EMBL, GenBank, DDBJ, which indicates that this is a new mutation. The mutation occurs in exon 1 of the transcript NM_019066.4, which may cause protein truncation or activate nonsense-mediated mRNA degradation, thus affecting the function of the protein product encoded by the gene.

## 5 Discussion

MAGEL2 is a patrilineal gene expressed in 15q11–15q13, a key region of the Prader-Willi domain, and their phenotypes are similar, so SYS is often misdiagnosed, at first, as PWS. The overlapping phenotypes of SYS and PWS mainly include growth retardation/mental retardation, neonatal dystonia, feeding difficulties and hypogonadism. With the expansion of the clinical cohort of individuals with truncated mutations in MAGEL2, the unique phenotypic characteristics of SYS became more obvious. This led to the subsequent renaming of this disease as SYS. The present study found that compared to PWS, SYS is more likely to develop joint contracture (88%), autism spectrum disorder (78%), and respiratory distress (71%),^[3]^ whereas the incidence of overeating and morbid obesity is lower in SYS than in PWS (35%–50%).^[4]^ Recently, it was reported that SYS patients show more severe mental retardation and growth retardation than PWS patients.^[3,5]^

**Figure 1:**
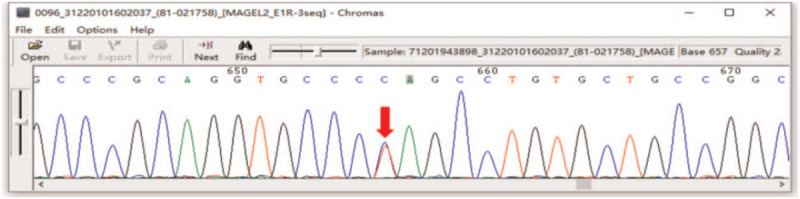
Gene sequencing map of a patient with SYS. Heterozygous variation of MAGEL2 gene c. 1687C > T (p.Q563X) on chromosome 15.

The patient reported in this study had postnatal language lag, growth retardation, no feeding difficulties, no body temperature instability, no gastroesophageal problems, no kyphosis/scoliosis, no seizures, no obvious dystonia and joint deformities. At present, the main manifestations are mild cognitive impairment (MoCA score 25), social fear, sexual development disorder, dyskinesia, obesity, short stature, smaller hands and feet than their peers. Therefore, we performed genetic sequencing on this patient. One heterozygous variation, NM_019066.4:c.1687C > T (p.Q563X), was found in exon 1 of the MAGEL2 gene by whole exon sequencing (Fig. 1).

In the review of MAGEL2-related disorders to date, we have summarised some frequent loci found so far (Table 1). However, due to the lack of sufficient samples of SYS, the phenotypic variation of many of these loci could not be very clear and needs further study. In summary, we found that the c.1996dupC mutation was the most common, followed by the c.1996delC mutation. Chen et al^[1]^ have described that the c.1996dupC mutation respiratory distress is more serious than other site mutations, while the c.1996del C mutation joint contracture is more common. So, we have reason to think that there is a genotype-phenotype correlation in SYS patients, which is consistent with the findings of McCarthy et al.^[1,3]^

**Table 1:** Relationship between partial gene variation sites and phenotype in patients with SYS.

Author	Variants	Phenotype
Gregory et al, 2019^16^	c.1996dupC, p.Q666Pfs∗47	Neonatal hypotonia, feeding difficulties, developmental delay/intellectual disability, multiple joint contractures, dysmorphic facial features, respiratory distress and multiple pituitary hormone deficiency
M. D. Fountain et al, 2017^17^		
X. Chen et al, 2020^[1]^		
J. McCarthy et al, 2018^[3]^		
Matuszewska et al, 2018^18^		
Igarashi et al, 2017^[8]^		
Gregory et al, 2019^16^	c.1996delC, p.Q666Sfs∗36	A lethal form of arthrogryposis, fetal akinesia, contractures, overlapping digits, rocker bottom feet, retro-micrognathia, gnatho-palatoschisis, multiple pterygia and bilateral congenital talipes equinovarus
M. D. Fountain et al, 2017^17^		
X. Chen et al, 2020^[1]^		
Kleinendorst et al, 2018^19^	c.1850G > A, p.Trp617∗	Excessive weight gain or obesity, hypotonia, apnea, pneumonia, dysmorphic facial features, spontaneous movements, hypotonia, laryngeal stridor and arthrogryposis
Tong et al, 2018^20^	c.1628delC(p.Pro543Leufs∗159)	Developmental delay, multiple joint contractures, muscular hypotonia, speech articulation difficulties, feeding difficulties in infancy, hypoglycaemia and dyspnoea
X. F. Chen et al, 2019^21^	c. 1640-1641delTT(p.Phe547fs)	Laggard language development and poor cognitive comprehension and dysmorphic facial features
Enya et al, 2018^22^	c.1912C > T, p.Gln638∗	Weak cry, hypotonia, poor feeding, respiratory failure requiring ventilator management, physical abnormalities including almond-shaped palpebral fissure and depressed nasal root, flattened philtrum, micrognathia, and camptodactyly involving the fingers
Enya et al, 2018^22^	c.3131C > A, p.Ser1044∗	Respiratory difficulty, generalised hypotonia, feeding difficulty, dysmorphic features included cleft palate, micrognathia, camptodactyly involving the fingers, congenital scoliosis and bilateral clubfoot
D. Hidalgo-Santos et al, 2018^23^	c.3019 C > T, p.Gln1007Ter	Distal contractures (equinovarus foot), hypotonia, feeding difficulties, ASD, digestive abnormalities and partial hypopituitarism with central hypothyroidism and growth hormone (GH) deficiency
Marbach et al, 2020^24^	c.2170 _ 2232dupp.(Ser724_ Ala744dup)	Feeding difficulties in infancy, developmental delay, learning difficulties, onset of hyperphagia in childhood, mild intellectual disability, sexual dysfunction, behavioural problems including bouts of aggression. chronic skin picking and uncontrolled eating
Matuszewska et al, 2018^18^	c.2894G > A, p.Trp965∗, hg19genome location ch15:023889996-D > T	Respiratory failure, muscular hypotonia, distal arthrogryposis, feeding difficulties. dysmorphic facial features include up-slanting palpebral fissures, bitemporal narrowing, high nasal bridge, low-set ears, prominent chin and micrognathia.

Why do Mutations of MAGEL2 cause so many phenotypes? We discussed the mechanisms of these 3 phenotypes which are quite different between PWS and SYS. In order to study its mechanism, a Magel2-deficient mice model was established based on the only pathogenic gene, Magel2. It was found that the mice had feeding impairment and learning, social, cognitive and communication problems. This finding was consistent with the phenotype of SYS.^[6]^ In terms of dyspnoea phenotype, Magel2 was highly expressed in abdominal wall muscle, diaphragm and other systems, and its loss led to low tension of the respiratory muscles, decrease in respiratory capability and lack of ventilation.^[7]^ Uncontrollable obesity was an important phenotype of the Magel2 mutation. It is well known that leptin is an important hormone related to obesity. So, is leptin involved? At present, there are 3 recognized mechanisms of obesity:

1. Deficiency in the ability to generate satiety signals in the feeding response,
2. Hypothalamic central dysfunction that controls energy homeostasis and
3. Abnormally high activation of the reward pathways in the brain by food-related stimuli.^[8]^

Leptin was involved in many of these pathways. There was evidence that Magel2 was involved in the leptin-POMC of the hypothalamus to maintain the energy balance of the body.^[9]^ Wijesuriya et al have found that MAGEL2 and Necdin connected leptin receptor (LepR) to the USP8-RNF41 ubiquitin complex through the LepR adapter protein, IRS4. Moreover, MAGEL2 changed the stability and intracellular localisation of LepR, USP8 and RNF41. The abundance of Necdin, LepR, Usp8, Rnf41 and ESCRT-0 complex protein Stam1 changed in the case of the hypothalamus of Magel2-Null mice. The loss of Magel2 disrupted the normal balance of expression, internalisation and degradation on the surface of LepR cells and resulted in leptin resistance and obesity.^[10]^ It was found that the related pathway of leptin in Magel2 was not only involved in the mechanism of obesity but also in the abnormal expression of autistic behavior. The binding of Magel2 and the membrane protein leptin receptor was involved in the transport of neurotransmitter receptors, which affected the development and functioning of the excitatory synapses regulated by dendritic remodelling in oxytocin neurons.^[11]^ Early correction of oxytocin improved the autism performance of Magel2-null mice. This also confirmed the mechanism of oxytocin involved in the autism phenotype.^[6,12,13]^ MAGEL2 is a member of the MAGE family of ubiquitin ligase regulators. MAGEL2 is a part of a multi-subunit protein complex consisting of MAGEL2, the TRIM27 E3 ubiquitin ligase and the USP7 deubiquitinating enzyme. TRIM27 has been implicated in several cellular and disease processes, including apoptosis, spermatogenesis, muscle atrophy and autism. USP7 is also mutated in a subset of children with similar phenotypes to those seen in PWS and SYS, providing further evidence that MAGEL2, with its binding partners, regulates biological pathways that are affected in patients with PWS and SYS.^[14,15]^ There is consensus among researchers about the abundant expression of MAGEL2 in the hypothalamus. Although the hypothalamus is very small, it controls a variety of important functional activities of the body, such as energy homeostasis, metabolic regulation and hormone secretion. The complexity of the hypothalamus determines whether the pathogenic mechanism of MAGEL2, that is, the pathogenic gene of SYS, is complex or not. To determine a more accurate and specific mechanism will require relevant researchers to spend more energy and time in this area.

## 6 Conclusion

We can suspect the likelihood of SYS according to the typical phenotype, but the final diagnosis needs to be based on genetic testing. Drawing on the continuous discovery of the SYS gene loci and the study of the correlation between mutation sites and phenotypes, our study has found that certain fatal phenotypes such as respiratory distress have a high incidence at individual sites, and early detection and timely intervention may prolong the life spans of patients. Therefore, in the case of patients for whom SYS is highly suspected, gene detection should be carried out as soon as possible.

## Author contributions

**Resources:** Xiuyun Jiang.

**Writing – original draft:** Yanjie Duan, Lu Liu.

**Writing – review & editing:** Xiujuan Zhang, Jin Xu, Qingbo Guan.
